# Exposure and power of TV food advertising during the COVID-19 pandemic in Brazil: a content analysis

**DOI:** 10.1186/s12889-024-17870-x

**Published:** 2024-02-26

**Authors:** Michele Bittencourt Rodrigues, Juliana de Paula Matos, Marina Oliveira Santana, Ana Paula Bortoletto Martins, Rafael Moreira Claro, Paula Martins Horta

**Affiliations:** 1 Department of Nutrition, Federal University of Minas Gerais (UFMG), Av. Alfredo Balena 190, 30130-100 Belo Horizonte, Minas Gerais Brazil; 2 Department of Nutrition, School of Public Health, University of São Paulo (USP), Av. Dr. Arnaldo, 715 - Cerqueira César, 01246-904 São Paulo, São Paulo Brazil; 3 Center for Epidemiological Research in Nutrition and Health, Department of Nutrition, School of Public Health, University of São Paulo (USP), Av. Dr. Arnaldo, 715 - Cerqueira César, 01246-904 São Paulo São Paulo, Brazil

**Keywords:** Food advertising, Television, COVID-19, Ultra-processed foods, Content analysis

## Abstract

**Background:**

With the COVID-19 pandemic and the restrictions imposed to contain the spread of SARS-CoV-2, the Brazilian population has increased the time spent at home and watching television (TV). Since food advertising exposure is a key driver of food choices, this study described the content of food advertisements (ads) on Brazilian TV during the COVID-19 pandemic.

**Methods:**

This is an exploratory study. A total of 684 h of TV programming comprised of three free-to-air channels and two pay-per-view channels was recorded from 06 a.m. to 12 a.m. for eight non-consecutive days in June 2020. A content analysis of all the food-related ads was carried out. The data collection process followed INFORMAS Protocol for TV food advertising monitoring.

**Results:**

The sample was composed of 7,083 ads, 752 (10.6%) of which were food-related and 487 (6.9%) were promoting ultra-processed foods. The content analysis indicated seven thematic categories, all of them with reference to the COVID-19 pandemic: brand and product differentials (79.8%); visual and sound effects (70.2%); thematic campaigns (56.0%); digitization (22.9%); convenience (16.5%); economic benefits (11.9%); and commensality and social interaction (6.1%). Ads content varied according to the day of the week, the time of the day, the length of the ad, and the channel type.

**Conclusions:**

The thematic of food advertising on Brazilian TV during the COVID-19 pandemic is aligned with the country’s health crisis context and varied during the programming.

## Background

Overweight is one of the most serious public health issues in the world. According to the World Health Organization (WHO) in 2016, more than 1.9 billion adults were overweight, with more than 650 million obese [1]. In Brazil, the prevalence of obesity in the adult population increased from 12.2% to 26.8% in less than 20 years (from 2003 to 2019). In the same period, the prevalence of overweight increased from 43.3% to 61.7% [2]. Sedentary behaviors, along with diets rich in ultra-processed foods are important factors in the etiology of overweight, obesity, and non-communicable diseases (NCDs) [1].

Food choice is a complex process that involves social and environmental factors, including exposure to advertising [3–6]. Integral to the food choice paradigm is food advertising, encompassing food and beverage advertising across various spaces where individuals engage, including television (TV) [7], digital environment (i.e., social media, websites, applications) [8], and outdoor mediums (i.e., public transport, posters, flyers, flags, banners, transit shelters, or benches, and billboards) [9–11] among others. Food advertising is currently focused on unhealthy foods frequently containing persuasive marketing strategies that stimulate the individuals’ desire for the advertised product and their loyalty to food brands [3–6]. Examples of these strategies are advertisement (ad) repetition, product demonstration, peer popularity appeal, celebrity endorsement, and premium offers [12, 13]. Notably, these advertising strategies have a significant impact, especially on children [14, 15]. This was confirmed by a study conducted on school-age children in Malaysia, which showed that children tend to prefer ads promoting unhealthy products like sugar-sweetened beverages, fast food, and snacks [14]. The study also highlighted that ads employing attractive branding, promotional characters, and toy giveaways were particularly effective [14].

Television (TV) remains one of the most dominant and pervasive forms of media globally, shaping cultural, social, and economic landscapes in diverse societies. Globally estimated 79% of households have at least one TV set, highlighting the widespread influence of this medium [16].

In Brazil, TV is the most used media by food companies to advertise their products, representing 71% of the advertising investments [17]. Additionally, a review of evidence over the last fifteen years on food and beverage marketing in Latin American countries emphasized TV as the primary advertising medium, with growing relevance for digital platforms, indicating the regional challenges related to unhealthy eating and the need for comprehensive research [18]. According to a monitoring study carried out in April 2018, the three most popular Brazilian TV channels promoted 14.2% of food-related ads, more than 90% of them containing at least one ultra-processed food and with one or more persuasive marketing strategies [19, 20]. In this country, 96.8% of the households have at least one TV set [21], and 25% of the population is exposed to this media for more than three hours a day [22].

The COVID-19 pandemic precipitated a significant surge in screen time worldwide, particularly among children [23]. As lockdowns and remote learning measures necessitated increased reliance on electronic media for education and entertainment [23]. A study conducted in Canada found that children's screen time doubled during the pandemic, with TV viewing accounting for a substantial portion of their daily activities [24]. In Brazil, this exposure increased by 1 h and 20 min after the beginning of the COVID-19 pandemic [25]. This unprecedented shift in media consumption patterns has raised concerns about the potential long-term implications for health and well-being, particularly in relation to sedentary behavior and its impact on physical activity levels and overall fitness [26, 27].

The analysis of food advertising during the COVID-19 pandemic presents an opportunity to illuminate discussions on multiple fronts. Restricting food advertising is a global imperative for health organizations, encouraging civil society organizations, academic researchers, and governments to actively monitor and tackle this issue [28–30]. Monitoring food advertising serves as a critical tool in comprehending the intricacies of consumer behavior, especially in the context of promoting healthy food choices and mitigating the risks associated with the prevalence of unhealthy food products. Robust monitoring initiatives facilitate the evaluation of advertising trends, empowering policy-makers and public health authorities to formulate evidence-based strategies for regulating the marketing of foods and beverages, particularly those targeting vulnerable populations such as children and adolescents. Consequently, providing results from diverse regions and countries offers valuable insights into the global landscape of food advertising practices, shedding light on the disparities in regulatory frameworks, advertising standards, and industry practices.

In Brazil, no single study has described the characteristics of TV food advertising during the COVID-19 pandemic. However, international literature indicates that the food industry took advantage of the high exposure of individuals to mass media during the pandemic ^(31)^ and innovated in advertising campaigns, using marketing strategies aligned with the context of social distancing and with strong appeals for corporate social responsibility. In other words, the food industry made use of a period marked by health, political, and economic crises to consolidate their market share and reach new consumers [31–34].

Considering the potential negative impacts of the expansion of TV food advertising after the COVID-19 pandemic, the lack of evidence in this field, and the importance of adopting new methodological approaches that can potentially serve as a framework for delineating advertising practices worldwide, this study aimed to analyze the thematic content of food-related ads in five TV Brazilian channels during the first phase (June 2020) of the COVID-19 pandemic.

## Methods

This is a cross-sectional study based on the Food Promotion Module: Food Marketing—Television Protocol [35] developed by the International Network for Food and Obesity/Non-Communicable Diseases Research, Monitoring and Action Support (INFORMAS).

TV broadcasts were recorded from three free-to-air and two pay-per-view channels, selected by their high audience in Brazil. The two pay-per-view channels are aimed at children, and the three free-to-air channels, although not exclusively dedicated to children, did include programming specifically designed for young audiences as part of their content offerings. Data were recorded by a media auditing service for eight non-consecutive days (four weekdays and four weekend days) randomly selected during June 2020, from 6 a.m. to 12 a.m. In total, 684 h of broadcasts were recorded (due to a recording failure, two days (36 h) of a pay-per-view channel were lost).

Ad information was extracted through a digital questionnaire using the software Epi Info ™ (version 7.2.2.6). All data extraction was conducted independently by pairs of trained researchers. All datasets were cross-checked to correct any mistakes (inter-coder reliability ranged = from 97.6% to 99.5%). Each non-food-related ad received a single generic code, while all food-related ads received a unique code. Food-related ads were then sub-classified in:

(i) Food or drink product – food company/brand (ultra-processed foods vs. non-ultra-processed foods);
(ii) Food or drink company or brand (no retailer) without food or drink product;
(iii) Food or drink retailer (supermarket or convenience store) with food or drink product;
(iv) Food or drink retailer (supermarket or convenience store) without food or drink product;
(v) Food or drink retailer (restaurant or takeaway or fast food) with food or drink product;
(vi) Food or drink retailer (restaurant or takeaway or fast food) without food or drink product [18].

The variables investigated for each food-related ad included: date of recording (i.e., day of the week or weekend); time of the day (i.e., morning, afternoon, or evening); ad length (i.e., less or equal 5 s, between 6 and 15 s, or between 16 and 30 s) and the channel type (i.e., free-to-air or pay-per-view).

We also carried out a thematic content analysis of the food-related ads according to Braun and Clarke [36] using the NVivo software (version 16.1) and Microsoft® Excel (version 16.59). The analysis followed the steps:

(i) Pre-analysis: two researchers carried out an initial analysis and took notes about the main thematic (advertising messages and appeals) that appeared in each ad;
(ii) Generation of initial codes: a researcher systematically coded the pre-analysis from the entire set of annotated data, grouping relevant data for each code. This step generated 42 initial codes;
(iii) Selection of categories: a researcher grouped the codes into potential categories, clustering all relevant data for each potential category. This step generated 12 initial categories;
(iv) Review of the categories: after a review of the coded data and categories, a thematic ‘map’ was generated for analysis and discussed among three researchers. This step resulted in a set of 7 thematic categories;
(v) Treatment of the categories: a researcher carried out a general analysis to inspect the features of each category. As a result, definitions and clear names were generated for each category;
(vi) Interpretation of results: two researchers independently analyzed ads according to the categories found in the previous steps;
(vii) Consistency analysis of the interpretation: all results were compared, and divergences were verified and corrected.

Also, an inspection was carried out for each ad aiming to identify content making any reference to the COVID-19 pandemic. Publicity references to staying at home, social distancing, minimal contact, boredom in quarantine, or some companies endorsing healthcare professionals, are examples of related mentions.

For the quantitative data analyses, the first step was to estimate the absolute number, proportion, and 95% confidence interval (CI) of each ad type and of each category identified in the content analysis (applies only to food-related ads). Then, we estimated the absolute number, proportion, and 95% CI of each ad type and content category stratified by COVID-19 reference, day of the week, time of the day, ad length, and channel type. Differences between values were considered statistically significant when their 95% CI did not overlap. All statistical analysis was carried out using the Stata statistical software package (version 18).

## Results

A total of 7,083 ads were recorded during 684 h of programming (= 2.07 ads per hour per channel). Food-related ads totaled 752 (10.6%; 0.22 food-related ads per hour per channel), most of them featuring a food or drink product by a food company or brand (*n* = 618; 8.7%), mainly ultra-processed foods (*n* = 487; 6.9%) (Table 1). Notably, 15% of the food-related ads directly referenced the COVID-19 pandemic. Among these ads, a significant number, 58 instances, involved food or drink products by a food company or brand (51.3%). Additionally, 48 instances highlighted the promotion of a food company or brand (42.5%) (Table 1).

**Table 1:** Type of the advertisements broadcasted from 6:00 am to 12:00 am on three free-to-air channels and two pay-per-view channels on Brazilian television in June 2020

Advertisement type	Total				COVID-19 pandemic reference			
	**n**	**%**	**95% CI**		**n**	**%**	**95% CI**	
***Food-related advertisements***	752	10.6	10.0	11.4	113	15.0	12.6	17.8
Food or drink product – food company/brand	618	8.7	8.1	9.4	58	51.3	42.2	60.4
Ultra-processed foods	487	6.9	6.3	7.5	48	42.5	33.7	51.7
Non-ultra-processed foods^a^	131	1.8	1.6	2.2	10	8.8	4.8	15.7
Food or drink company or brand (no retailer) without food or drink product	23	0.3	0.2	0.5	13	11.5	6.8	18.8
Food or drink retailer (supermarket or convenience store) with food or drink product	57	0.8	0.6	1.0	10	8.8	4.8	15.7
Food or drink retailer (supermarket or convenience store) without food or drink product	20	0.3	0.2	0.4	5	4.4	1.8	10.1
Food or drink retailer (restaurant or takeaway or fast food) with food or drink product	22	0.3	0.2	0.5	15	13.3	8.2	20.8
Food or drink retailer (restaurant or takeaway or fast food) without food or drink product	12	0.2	0.1	0.3	12	10.6	6.1	17.8
***Non-food-related advertisements***	6,331	89.4	88.6	90.1	NA	NA	NA	NA
**Total**	7,083	100.0						

*NA* Not applicable—Content analysis was not conducted for advertisements unrelated to food or those lacking food-related content. Consequently, it was impossible to assess their reference to the COVID-19 pandemic

^a^Includes fresh and minimally processed foods, culinary ingredients, processed foods and nutritional supplements

The thematic content analysis of food-related ads showed seven thematic categories: *(i)* ‘brand and product differentials’: ads highlighted the product, production process, brand, and/or ingredients; *(ii)* ‘sound and visual effects’: eye-catching and dynamic ads using visual and sound effects that aroused viewers’ emotions and feelings; *(iii)* ‘thematic campaigns’: ads with specific thematic campaigns such as special dates, aimed at children, care and well-being, and social responsibility; *(iv)* ‘digitization: ads that expanded the viewer’s contact with the brand, through new digital technologies; *(v)* ‘convenience’: ads with appeal of practicality and ease of consumption or buying the product; *(vi)* ‘economic benefits’: ads with the appeal to economic benefits, such as discounts, loyalty programs, among others; and *(vii)* ‘commensality and social interaction’: ads with references to eating together and encouraging social interaction, both online and physically. Each of these thematic is described in detail and exemplified in Table 2.

**Table 2:** Description of thematic categories identified in food-related advertisements on three main free-to-air channels and two pay-per-view channels on Brazilian television in June 2020

Thematic categories	Description	Example with no COVID-19 pandemic reference	Example with COVID-19 pandemic reference
Brand and products differentials	Ads that praised the product, production process, brand, and/or ingredients (e.g., national or international product). They could also reinforce the quality and sophistication of the advertised product. Other appeals were related to denominations such as “the best” and highlight for product launches	The image shows a piece of meat (pork ribs) with a phrase highlighting the ingredients: “The best ingredients”	The image shows an ad for a throat drops, featuring a 'doctor' asserting the necessity of protecting the family during the present times while highlighting the brand's long-standing tradition in this regard
Sound and visual effects	Eye-catching, dynamic, and funny ads that triggered emotions and feelings using special effects such as those exploring sensorial, visual, and hearing characteristics	The image shows a chocolate bar with the effect of melting chocolate	The image shows an ad for a beer brand, asserting that the 'new normal' would not be an empty beach, and emphasizing the collective effort required to return to social gatherings and leisure activities. The ad effectively utilizes auditory and visual effects, incorporating sounds like the opening of the beer bottle and the integration of the beverage with the sea, thereby establishing a strong association with the promoted product
Thematic campaigns	Ads with a defined thematic for *(i)* special dates, such as Valentine’s Day; *(ii)* “for kids”, ads that had childish language, children’s characters, excess of colors and illustrations in drawings; *(iii)* care and well-being, ads that reinforce care, for oneself or for others (e.g., women taking care of children), in addition to claims for well-being (e.g. provides energy) and health (e.g., benefits to staying healthy); and *(iv)* social responsibility, ads showing solidarity campaigns, financial aid and social actions to benefit the population	The first image shows a couple in the background and a phrase referring to the Valentine’s Day. The second image shows some dairy products from a brand, with children’s characters in the front of pack. The third image shows a mother and her daughter in the background with the message written in the main plan “For you who take care”. The fourth image shows the logo of a solidarity campaign of a meal delivery brand that donates a meal to socially vulnerable people for each order received	The image shows an advertisement for a soft drink company, portraying a deserted cityscape while emphasizing the company's solidarity with society, affirming the collective resilience necessary to overcome this health crisis
Digitization	Ads that expanded the individual’s experience by using a digital element, such as a QR Code or a link to a website or app	The image shows an electronic address (searalivefest.com.br) and a QR Code that directs the user to a company website. In the background, two people are watching a “Live Fest” on TV	The image shows an ad for a beer company, featuring a QR code and the company's app, enabling viewers to conveniently order the product and have it delivered safely to their homes, facilitating continued virtual meetings with friends
Convenience	Ads that presented appeal for practicality and ease of consuming or buying the product	The image shows a bowl of Mac and Cheese in the background and a sentence in the foreground that indicates that this dish only takes five minutes to become creamy, as shown in the image	The image shows an ad, emphasizing that despite certain closures during the health crisis, the company's burgers and meals remained easily accessible through a streamlined and contactless process, facilitated by quick and convenient drive-thru orders
Economic benefits	Advertising appeals to economic benefits (e.g., promotions, discounts, free delivery, limited time, benefits for new customers, loyalty programs, price, etc.)	The image shows phrases indicating economic benefits. On the left side we see a 15% discount on a product line. On the Smartphone screen there is a phrase that indicates exclusive discounts on the App used by the company	The image shows an ad for a food delivery app company, featuring an economic appeal from a campaign encouraging viewers to place their first order for just R$ 5 ($1)
Commensality and social interaction	Ads that praised eating together (e.g., with family and/or friends). They could also stimulate social interaction, both online and physically regardless of whether individuals were sharing food or meal	The image shows a family having lunch together	The image shows an ad for a beer company, featuring friends sharing meals together through a virtual call

Food-related ads that addressed the ‘brand and product differentials’ and ‘sound and visual effects’ were the most frequent: 79.8% (95% CI: 76.7; 82.5) and 70.2% (95% CI: 66.8; 73.4), respectively. Subsequently, the ‘thematic campaigns’ were identified in 56.0% of the ads (95% CI: 52.4; 59.5) and ‘digitization’ in 22.9% (95% CI: 20.0; 26.1). The other categories represented less than 20% of the ads. Food-related ads also made reference to the COVID-19 pandemic in all the seven thematic categories. However, this reference was more common in ads that addressed ‘thematic campaigns’ (73.4% – 95% CI: 64.5; 80.9), ‘brand and product differentials’ (69.9% – 95% CI: 60.8; 77.6), and ‘sound and visual effects’ (54.0% – 95% CI: 44.7; 62.9) (Table 3).

**Table 3:** Thematic categories in food-related advertisements and mentions of the COVID-19 pandemic on three free-to-air channels and two pay-per-view channels on Brazilian television in June 2020

**Thematic categories**^a^	**Total**				**COVID-19 pandemic reference**			
	**n**	**%**	**95% CI**		**n**	**%**	**95% CI**	
Brand and products differentials	600	79.8	76.7	82.5	79	69.9	60.8	77.6
Sound and visual effects	528	70.2	66.8	73.4	61	54.0	44.7	62.9
Thematic campaigns	421	56.0	52.4	59.5	83	73.4	64.5	80.9
Digitization	172	22.9	20.0	26.1	32	28.3	20.8	37.3
Convenience	124	16.5	14.0	19.3	31	27.4	20.0	36.4
Economic benefits	89	11.9	8.4	12.8	12	10.6	6.1	17.8
Commensality and social interaction	45	6.1	4.6	8.1	6	5.6	2.5	11.8

^a^Thematic categories identified in food-related advertisements (*n* = 752). An advertisement can be classified in more than one thematic category

Table 4 shows the frequencies of the thematic categories present in the food-related ads stratified by the day of the week and the time of the day the advertising was broadcasted. In the first case, ‘brand and products differentials’ was more common during the week (81.8%) than on the weekend (76.5%). On weekends ‘sound and visual effects’ was the second promoted category (76.1%). Also, ‘digitization’ thematic was more frequent in ads broadcasted on weekends (24.2%) than during the week (18.9%). ‘Commensality and social interaction’ was the least frequent thematic, both on weekends (7.0%) and during the week (8.3%).

**Table 4:** Thematic categories in food-related advertisements stratified by day of the week and time of the day on three free-to-air channels and two pay-per-view channels on Brazilian television in June 2020

Thematic categories	Day of the week								Time of the day											
	**Weekday**				**Weekend**				**Morning**				**Afternoon**				**Evening**			
	**n**	**%**	**95% CI**		**n**	**%**	**95% CI**		**n**	**%**	**95% CI**		**n**	**%**	**95% CI**		**n**	**%**	**95% CI**	
Brand and products differentials	382	81.8	78.0	85.0	218	76.5	71.2	81.1	153	81.8	75.6	86.7	209	82.0	75.6	86.7	238	76.7	71.7	71.7
Sound and visual effects	311	71.0	67.4	74.4	217	76.1	70.8	80.7	133	71.1	64.3	77.1	186	72.9	67.1	78.0	209	67.4	61.9	61.9
Thematic campaigns	264	57.1	53.3	60.9	157	55.1	49.2	60.7	104	55.6	48.4	62.6	159	62.3	56.2	68.0	158	51.0	45.4	56.5
Digitization	103	18.9	16.0	22.1	69	24.2	19.6	29.5	47	25.1	19.4	31.8	56	22.0	17.3	27.5	69	22.2	18.0	27.5
Convenience	78	17.5	14.7	20.6	46	16.1	12.3	20.9	32	17.1	12.3	23.2	39	15.3	11.4	20.3	53	17.1	13.3	13.3
Economic benefits	60	10.2	8.1	12.8	29	10.2	7.1	14.3	18	9.6	6.1	14.8	32	12.6	9.0	17.2	39	12.5	9.3	16.8
Commensality and social interaction	22	7.0	5.2	9.2	23	8.3	5.6	12.2	9	4.9	2.6	9.2	14	5.7	3.4	9.3	22	7.3	4.8	10.8

According to the time of the day in which the ads were broadcasted, ‘brand and product differentials’, ‘sound and visual effects’, and ‘thematic campaigns’ were the most frequent thematic categories in the three time periods. Ads classified in ‘thematic campaigns’ category were more broadcasted in the afternoon (62.3%) than in the evening (51.0%). For the other categories, no other differences were noted (Table 4).

Table 5 shows the frequencies of thematic categories stratified by ad length and channel type. In the first case, the ‘brand and products differentials’ and ‘sound and visual effects” were the most frequent categories in the three-length ad’ groups. Additionally, the thematic campaigns were also frequent among both the ‘between 6 and 15 s’ and ‘between 16 and 30 s’ groups. In contrast, no ad with ‘less or equal 5 s’ was classified in the economic benefits’, and ‘commensality and social interaction’ categories. While the categories ‘brand and products differentials’ and ‘sound and visual effects’ remained consistent across all three ad length categories, the remaining thematic categories exhibited a notably lower frequency in ads ‘less or equal 5 s’ compared to those ‘between 6 and 15 s’ or ‘between 16 and 30 s’.

**Table 5:** Thematic categories in food-related advertisements stratified by advertisement length and channel type on three free-to-air channels and two pay-per-view channels on Brazilian television in June 2020

Thematic categories	Advertisement length												Channel type							
	**Less or equal 5 s**				**Between 6 and 15 s**				**Between 16 and 30 s**				**Free-to-air**				**Pay-per-view**			
	**n**	**%**	**95% CI**		**n**	**%**	**95% CI**		**n**	**%**	**95% CI**		**n**	**%**	**95% CI**		**n**	**%**	**95% CI**	
Brand and products differentials	93	73.8	65.4	80.7	256	87.7	83.3	90.9	251	75.1	70.2	79.5	539	80.4	77.8	83.7	61	70.9	60.4	79.6
Sound and visual effects	83	65.9	57.2	73.6	203	69.5	64.0	74.5	242	72.4	67.4	77.0	459	68.9	65.3	72.3	69	80.2	70.4	87.4
Thematic campaigns	17	13.5	8.6	20.6	183	62.7	56.9	68.0	221	66.2	60.9	71.0	369	55.4	51.6	59.1	52	60.5	49.8	70.2
Digitization	1	0.7	0.1	5.4	63	21.6	17.2	26.6	108	32.3	27.5	37.5	141	21.1	18.2	24.4	31	36.0	26.6	46.6
Convenience	5	4.0	1.7	9.2	71	24.3	19.7	29.5	48	14.4	10.9	18.6	114	17.1	14.4	20.1	10	11.6	6.6	20.2
Economic benefits	0	^a^			47	16.1	12.3	20.7	42	12.6	9.4	16.6	79	11.8	9.6	14.5	10	11.6	6.4	20.2
Commensality and social interaction	0	^a^			15	5.1	3.1	8.4	30	9.5	6.7	13.3	45	7.0	5.2	9.2	0	^a^		

^a^No case

According to the channel type in which the ads were broadcasted, the ‘brand and products differentials’ thematic was more frequent in the ‘free-to-air’ programming (80.4%) than in ‘pay-per-view’ channels (70.9%). In the ‘pay-per-view’ channels, on the other hand, the most frequent thematic category was ‘sound and visual effects’ (80.2%). Also, ads classified in the category of ‘commensality and social interaction’ were more commonly seen in the ‘free-to-air’ channels (7.0% – 95% CI: 5.2; 9.2) than in the ‘pay-per-view’ channels (0% – 95% CI: no variation on cases). In contrast, ‘digitization’ thematic was more used in ads broadcasted in the ‘pay-per-view’ programming (36.0% – 95% CI: 26.6; 46.6) than in the ‘free-to-air’ channels (21.1% – 95% CI: 18.2; 24.4).

## Discussion

This study analyzed the thematic content of food-related ads on five TV channels during the first phase of the COVID-19 pandemic in Brazil. This was a period when Brazilians were encouraged to stay at home and were more exposed to TV programming. The findings revealed that food and beverage companies predominantly promoted ultra-processed foods, employing diverse content and references to the COVID-19 pandemic to engage viewers and encourage them to purchase their products. This content was distributed differently during the programming, according to the day of the week (with more informative ads on weekdays and more relaxed ads on weekends), the time of the day (with ads targeted at a specific audience according to the time of day), the length of the ad (with longer ads including a mix of themes), and the TV channel type (with pay-per-view channels targeting their content for children). These observations suggest that food companies strategically align their advertising messages with the audience profile, which can strongly influence the purchase decision.

A global overview of food advertising content on 22 countries’ TV programming showed that food-related ads accounted for 23% of all ads. The study also found four times more ads for foods and beverages with inadequate nutrients than for those with adequate nutrient content [7]. In Brazil, previous analyses have demonstrated a range in the prevalence of food-related ads in TV programming, spanning from 10.2% to 14.2% [19, 37–39]. Notably, multiple factors specific to each country may influence the prevalence of food advertising, thereby contributing to the observed differences in advertising proportions [40]. Possible explanations for the relatively lower occurrence of food advertising in Brazil compared to other countries could include *(i)* cultural preferences and eating habits that emphasize less food advertising or prioritize alternative marketing strategies, *(ii)* market dynamics and competitive scenarios in the Brazilian food industry, potentially leading to reduced emphasis on TV ads for food products, and *(iii)* differences in the marketing approaches adopted by the Brazilian advertising industry, focusing on a diverse range of platforms for content dissemination. Despite the lower occurrence of this type of ad on Brazilian TV in comparison to the global data [7], the Brazilian studies’ results corroborate the high presence of unhealthy food in TV programming [19, 37–39]. This is a concerning scenario from a public health point of view, especially during a health crisis when excessive consumption of unhealthy foods can increase the risk of COVID-19 aggravation [41].

The impact of food marketing on food consumption and health outcomes depends on both, “exposure” and the concept of “power” [42], which involves the utilization of various creative strategies. Previous research on the influence of advertising has primarily focused on food ads targeting children [42], examining the presence of objectively determined persuasive marketing strategies [43–45]. Our study introduces an innovative approach by analyzing advertising characteristics across a comprehensive range of ads, not limited to those targeting children, and employing thematic analysis instead of a structured analysis of individual marketing strategies within the ads. The thematic analysis allows for a more flexible research methodology, facilitating the identification of key characteristics through a comprehensive examination of the marketing strategies and themes employed. This approach enables us to identify similarities, differences, and recurring patterns within the ads, offering valuable insights and interpretations of social and behavioral implications. Ultimately, it provides a comprehensive and effective analysis of how the food industry employs marketing to shape consumer behavior [36].

Our findings showed three main thematic categories that were particularly prominent in TV food advertising in Brazil during the COVID-19 pandemic: ‘brand and product differentials’; ‘sound and visual effects’; and ‘thematic campaigns’. The first thematic category explored brand or product features, such as the production process (e.g., slowly roasted), the ingredients used (e.g., 100% sustainable cocoa), and the brand or product's positioning in the market (e.g., the best). When references to COVID-19 were made, the brand aligned itself with consumers, offering products that promote pleasure and well-being during the challenging period we were all experiencing.

The elements of ‘Brand and product differentials’ are commonly recognized in the marketing field as the “communication of the brand image” and have a more informative purpose. This type of content creates an idea of uniqueness, which is related to how much the brand and its products differ from competitors in the customers’ minds [46–48]. Therefore, when communicating their image, the brands need to target a wider audience, which could justify the fact that this type of content has been broadcasted more on ‘weekdays’ and ‘free-to-air channels’, as observed in our results.

The second most frequent thematic category among the food-related ads was ‘Sound and visual effects’. Using sensory resources (e.g., ingredients mixing to form a delicious product and very creamy cheese wrapping a pasta dish) that engage the consumers’ sensory senses and affect their perception, judgment, and behavior, food companies make the product attractive and reaffirm the qualities of both the brand and the product [49, 50]. In the context of COVID-19, ads classified in this category often employed emotionally evocative soundtracks, alluding to moments of uncertainty. Additionally, some ads associated sound with visual effects, for example, in the case of an ad that simulated a sea wave bringing a bottle (i.e., the beverage that was being advertised) and associated it with happy moments with friends on a summer day at the beach. These sound and visual effects could have been used in association to reinforce the message that happy days would come back.

A survey with seventy-two participants of two nationalities (Chinese and Danish) demonstrated that specifically tailored music can direct consumers’ visual attention to specific foods, suggesting that the brain does indeed integrate multiple streams of sensory information during decision-making [51]. Furthermore, initial research has suggested that certain sound effects can aid in enhancing viewers' recollection of an advertised product and increase their attentiveness to the ad [52, 53]. Visual effects can effectively convey persuasive messages, swiftly capturing viewers' attention and stimulating consumer demand [54]. For example, an ad showing a chocolate bar melting generates the sensation of dynamism and movement and the spectator continues to remember the image in movement. Consequently, this strategy encourages consumers to spend more time engaging with the ad [50].

Given these characteristics, it is understandable why ‘sound and visual effects’ were more frequent in the ads broadcasted on ‘pay-per-view’ channels, which primarily target children in Brazil. A review of marketing strategies employed by food and beverage companies for communicating with children on TV showed the frequent use of visual effects (e.g., animation, fast cutting, slow motion, dynamic images) are among the five more commonly used strategies [55]. ‘Sound and visual’ effects were also more common on weekend days when individuals have different eating behaviors in relation to the days of the week. Consumers are more likely to consume more palatable meals and foods on Saturdays and Sundays that escape the usual routine. Brazilians’ energy intake and ultra-processed food consumption are higher on weekends compared to weekdays [56].

‘Thematic campaigns’ was the third most common thematic category in the food-related ads broadcasted on Brazilian TV during COVID-19. Thematic advertising campaigns are intended to communicate with specific audiences. In the context of our study, one of these audiences is youth and adults. We found some ads with emotional appeal focused on Valentine’s Day (celebrated in Brazil on June 12) that associated the consumption of a food product with the celebration of couples and happy moments. The ads emphasized how couples were facing this moment together and promoted special events for Valentine’s Day as “Brahma Live”, with performances by famous singers in Brazil sponsored by a beer brand. Thus, ads created a new way of communication that uses storytelling to establish emotional connections with customers. According to Elías Zambrano et al. (2018), storytelling is a powerful marketing tool that enhances brand visibility by creating superior advertisement production quality and heightened sensitivity. This is especially true for television advertising, where the effective use of storytelling techniques sets certain brands apart from others [57].

Furthermore, some ads in ‘thematic campaigns’ targeted women directly, emphasizing product uniqueness and special production methods to promote health benefits and overall well-being. Within the context of the COVID-19 crisis, one ad depicted the human throat as a common site for SARS-CoV-2, suggesting the use of certain drops as a preventive measure. A document from the Global Health Advocacy Incubator (GHAI) has already pointed out the claims used by the food industry that some foods or beverages act as a booster for the immune system in the era of COVID-19 [32]. These claims create a misleading "halo of health" around low-nutritional-quality food and beverage products, potentially leading to misinformed dietary choices [58], particularly concerning individuals vulnerable to the fear of illness during a pandemic.

Engagement with the children’s audience was also noted within the ‘thematic campaigns’ category. We noticed an array of aspects, such as auditory appeals (e.g., children’s language and jingles); visual appeals (e.g., graphic design, animation); appeal to fun, taste, fantasy, adventure, or action; and the use of promotional characters. The companies also directed the ads to families, especially to mothers. These messages used images of children and women and were related to the act of caring and the concern of feeding children with quality products; through emotional appeals, they were able to involve the viewer. This thematic category was predominantly broadcasted on ‘pay-per-view’ channels and in the afternoon. While specific data on the peak TV viewing times for different age groups in Brazil is not publicly available, it is common for Brazilian children to spend several hours watching TV, particularly as they attend school part-time, either in the morning or afternoon. Thus, the afternoon can be considered a strategic time for food companies to directly target children [59]. Additionally, due to the limited children's programming on free-to-air channels, mostly restricted to a few hours during weekend mornings, 'pay-per-view' channels have become a primary attraction for children, thereby becoming a crucial target for the food and beverage industry's advertising efforts [19, 20].

Social responsibility content represented the fourth type of appeal included in the ‘thematic campaigns’ category. In this category, companies produced ads that presented solidarity campaigns, such as one major food delivery company that encouraged customers to order meals through their delivery app, with a promise to donate a meal to support vulnerable communities for every order placed. Additionally, another strategy of the companies was to present security measures that the companies were adopting to protect their employees and customers. This approach has been named COVID-washing by other authors and has been reported on food industry content posted on social media platforms from Australia, the United States, Uruguay, Argentina, Bolivia, and Guatemala [34, 60–63]. In the context of the pandemic, food industries disseminated symbolic information about their contributions to health and well-being. Companies use this approach to build an image of a responsible and committed company, as consumers prefer food brands that care about society, the environment, and/or their employees [47, 48].

The category ‘Digitalization’ emerges as a thematic category in 1 out of every 5 TV ads. Although TV is still one of the main channels of communication, the digital era, characterized by the significant influence of the Internet and digital technologies, has also promoted a change in advertising [64]. Currently, advertising content is becoming increasingly interactive, spanning multiple communication channels, enabling personalized content and a more extensive reach to diverse audiences [65]. This was observed in our study through the ads that presented elements of ‘digitization’, such as the presence of QR codes and links.

The category ‘digitization’ was more frequent in pay-per-view channels. However, the digital elements were not directed to children (for example, links to download games), as previously shown by other studies [55]. In our study, the main links were aimed at the family, when informing about a product or providing offers from a supermarket. In the context of COVID-19, links were also used to direct the viewer to websites containing information on the protective measures adopted by companies (e.g., food delivery companies) to prevent the transmission of SARS-CoV-2 among their employees and consumers. Moreover, companies shared links and/or QR codes to facilitate secure and convenient online purchases.

In Brazil, online purchases grew during the pandemic, and a food delivery market monitoring survey showed that 68% of Brazilian users (*n* = 1,500) of these platforms use the service on weekends [66]. This may justify that ads containing digitization elements are served more on weekends. Additionally, the growing prominence of e-commerce, coupled with the rising usage of smartphones for food purchases among Brazilians, further supports the prevalence of ads featuring digital elements, especially during weekend broadcasts.

Our findings also revealed that the thematic categories ‘convenience’ and ‘economic benefits’ represented a lower percentage of food-related ads. According to our analysis, this observation may be attributed to the innovative approaches adopted by the food industry in their advertising strategies. Consequently, while the literature suggests that convenience and affordability are commonly employed appeals in ads for ultra-processed foods [20], these two appeals may have been replaced by more casual and interactive messages, and with fewer commercial elements, to reach new customers.

In Brazil, food advertising during the COVID-19 pandemic moved away from strategies that emphasize convenience and price, with a deliberate alignment to the context of social distancing. Notably, companies predominantly adopted empathetic and solidarity-oriented messages, fostering a closer rapport between the brand and the consumer, reflecting a growing trend of humanizing the brand [33]. This strategic shift signifies the food industry's effort to self-promote through persuasive strategies, particularly during times of heightened vulnerability. Furthermore, the advertising strategies adopted by this industry aimed to generate market demand, influence the consumer purchase decision, and increase their loyalty to the supplier brands [33].

The last thematic category identified in this content analysis was ‘commensality and social interaction’. The term “commensality” refers to the positive social interactions that are associated with people eating together [67]. Eating together is an essential aspect highlighted in the Dietary Guidelines for the Brazilian population [68]. The food industry may have appropriated recommendations by the Brazilian dietary guidelines and included them in the ads, although the Ministry of Health document advocates a diet rich in natural foods, and nutritionally balanced meals, and promotes socially and environmentally sustainable food systems [68]. This appropriation has taken place at strategic moments, such as on weekends, when socialization appeal is already more frequent in TV ads, and on ‘free-to-air channels’, aiming to communicate with a more diversified audience.

Besides the day of the week, the time of the day, and the type of channel, another interesting feature that can help to understand how food advertising influences consumer choices is the variety of thematic categories used according to the duration of advertising. We observed that ads with longer duration had a greater diversity of thematic categories. This suggests that food companies leverage longer ad durations to incorporate a mix of themes. In our study, at least five of the seven identified thematic categories were most commonly featured in longer-duration ads. Longer-duration ads make it possible to use different appeals and communicate to different audiences. For example, an ad lasting ‘between 16 and 30 s’ contained appeals on commensality and social interaction in association with a QR code (i.e., digitization element), which allowed the consumer to know more about the advertised products. A potential inference is that longer ads have greater ‘power’, resulting in a stronger impact on consumers' consumption choices.

Our study contributes to the characterization of food advertising on Brazilian TV by employing an expanded and in-depth thematic content analysis of the power of strategies, a methodology not adopted by previous studies on the subject in the country. Our findings can help to understand how these thematic categories can potentially influence the consumer and how the broadcast of these ads in a non-random way during programming can be strategic for companies. Moreover, the results reinforce the need to continue to measure, monitor, and combat the use of persuasive techniques in food advertising. To address these issues, we propose some paths for future research and policy development. First, our results point to the need to carry out new studies on the subject, especially studies with an experimental design to understand how individuals are influenced by different patterns of marketing strategies. Second, it is necessary to impose regulatory limits that align with the nature of the identified marketing strategies, mainly on those that exploit the vulnerability of minors and lead consumers to errors on the profile of the advertised foods. Third, it is necessary to educate and empower the population about the nature of the persuasive strategies used in food ads and the harmful effects associated with the excessive consumption of ultra-processed foods.

Regarding the study limitations, first, thematic analysis is a subjective type of analysis. Therefore, data collection was carried out by two trained researchers, and any divergence was discussed and cross-verified with a third researcher to standardize all information and minimize subjectivity. Additionally, after completing the ‘treatment of the categories’ (step v of the thematic content analysis), the authors re-evaluated each ad to verify if all the thematic categories were correctly assigned to the ads. Second, the results represent food advertising during the first phase of the COVID-19 pandemic in Brazil. The thematic content of food ads may have changed as the pandemic advanced in the country.

## Conclusion

The food and beverage companies employed diverse thematic categories and references to the COVID-19 pandemic to promote ultra-processed foods, engage with viewers, and stimulate product purchases. This promotional content was broadcast unevenly throughout the programming schedule, according to the day of the week, the time of day, the duration of the ad, and the type of TV channel. This demonstrates that food advertising on Brazilian TV during the COVID-19 pandemic is aligned with the context of the country’s health crisis and exhibits characteristics within programming.

## Data Availability

The datasets generated and/or analyzed during the current study are not publicly available but are available from the corresponding author on reasonable request.

## Abbreviation

AD: Advertisement
CI: Confidence interval
GHAI: Global Health Advocacy Incubator
INFORMAS: International Network for Food and Obesity/Non-Communicable Diseases Research, Monitoring and Action Support
NCDs: Non-communicable diseases
TV: Television
WHO: World Health Organization
